# LIM domain‐containing protein Ajuba inhibits chemotherapy‐induced apoptosis by negatively regulating p53 stability in colorectal cancer cells

**DOI:** 10.1002/1878-0261.13421

**Published:** 2023-04-03

**Authors:** Beihui Xu, Qi Li, Jianjun Zhang, Fuxiang Chen

**Affiliations:** ^1^ Laboratory Medicine, Ninth People's Hospital Shanghai Jiao Tong University School of Medicine China; ^2^ State Key Laboratory of Drug Research, Shanghai Institute of Materia Medica Chinese Academy of Sciences China; ^3^ Department of Oral and Maxillofacial‐Head & Neck Oncology, Ninth People's Hospital Shanghai Jiao Tong University School of Medicine China; ^4^ Faculty of Medical Laboratory Science Shanghai Jiao Tong University School of Medicine China

**Keywords:** Ajuba, apoptosis, chemoresistance, colorectal cancer, p53

## Abstract

LIM protein‐domain containing protein Ajuba (encoded by *AJUBA*) functions as a scaffold protein to regulate protein–protein interactions, signalling transduction and genes transcription. *AJUBA* expression is higher in colorectal cancer (CRC) tissues than normal tissues, but its specific molecular function in CRC progression is still not very clear. Here, we found that, in CRC cancer cell lines, overexpression of *AJUBA* decreased p53 levels, whereas knock‐down of *AJUBA* significantly increased p53 levels. Although the presence of Ajuba did not influence p53 transcription, it formed a complex with p53 and MDM2 to promote the degradation of p53. *AJUBA* overexpression reduced the sensitivity of cancer cells to chemotherapeutic drugs and vice versa. In addition, chemotherapeutic drugs significantly induced *AJUBA* expression, which was largely dependent on the presence of p53. Therefore, Ajuba formed a negative feedback loop to regulate p53 expression and activity. In conclusion, as a novel p53‐negative regulator, Ajuba inhibits the apoptosis of CRC cells induced by chemotherapeutic drugs and it may be a new therapeutic target for CRC treatment.

## Graphical abstract

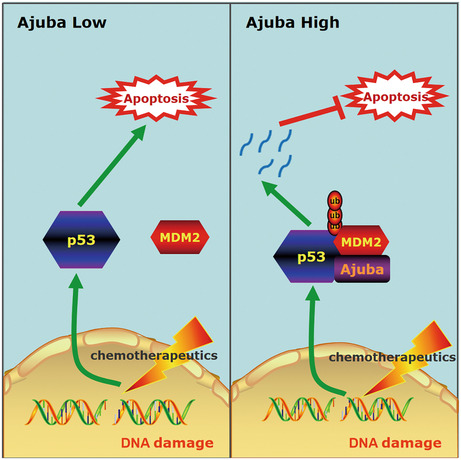

**Abbreviations**
5‐FU: 5‐flurorouracil
ADR: adriamycin
CHX: cycloheximide
co‐IP: co‐immunoprecipitation
CRC: colorectal cancer
DBD: DNA‐binding domain
ETO: etoposide
IHC: immunohistochemistry
NES: nuclear export sequences
NLS: nuclear localization sequences
Oxa: oxaliplatin

## 1 Introduction

Colorectal cancer (CRC) has become one of the most common digestive tract malignancies worldwide. Its morbidity and mortality occupy the third and second position in malignant tumours, respectively [1]. Because the lack of specific symptom, in most cases, the tumour has developed into its late stage at the time of diagnosis. Although new therapeutic strategies are emerging, chemotherapy drugs including 5‐FU and oxaliplatin still play important roles in the treatment of CRC. Unfortunately, many CRC patients developed with primary or acquired resistance to these chemotherapy drugs [2, 3]. Improving or predicting chemotherapy sensitivity is important for CRC patient treatment.

Ajuba belongs to a family of proteins characterized by LIM‐domain and is involved in numerous biological processes. It exerts its function as a scaffold protein，acting as a co‐regulator of transcription factors in the nucleus or regulating protein–protein interaction and signalling transduction in the cytoplasm [4, 5]. Ajuba protein is characterized with three tandem LIM domains at its C terminus that primarily mediates protein–protein interaction. Ajuba contains both nuclear localization sequences (NLS) and nuclear export sequences (NES);thereby, it can shuttle between nucleus and cytoplasm [5, 6]. In nucleus, Ajuba functions primarily as a transcriptional co‐regulator, such as a corepressor of Snail [7, 8] or a coactivator of PPARγ [6] and ERα [9]. In cytoplasm, Ajuba regulates many signal transduction pathways, including Hippo signalling [10], Notch signalling [11, 12] and Wnt signalling [13]. In CRC cells, Ajuba inhibits IFNγ‐induced cell death in CRC cells through directly suppressing JAK1‐STAT1 network [14].

Transcriptional factor p53 is a critical sensor for various stress including DNA damage induced by chemotherapy drugs, and it transcriptionally regulates many target genes expression and mediates cell cycle arrest and cell apoptosis [15]. In unstressed cells, p53 levels are kept low due to its polyubiquitination by the E3 ubiquitin ligase MDM2. In response to DNA damage induced by chemotherapeutic drugs, the N‐terminal region of p53 is phosphorylated by many kinases including ATM/ATR/DNA‐PK and CHK1/2. The phosphorylation of p53 at ser15 and ser20 results in its disassociation from MDM2 and eventually p53 is stabilized. P53 stabilization triggers a series of cellular responses including cell cycle arrest, DNA‐damage repair and apoptosis [15, 16]. In addition, MDM2 expression can be directly induced by p53, and this negative feedback loop comprise the primary p53 dynamic regulation. Many proteins are reported to interact with p53 and/or MDM2 and further refine or complicate the dynamic regulation of p53 [17]. In colorectal cancer, loss of p53 function plays a critical role in colorectal tumourigenesis, and p53 is also crucial for chemotherapeutic drug response, and loss of p53 abolishes the apoptotic response to 5‐flurorouracil (5‐FU) in CRC cells [18, 19, 20]. P53 is one of the most frequently mutated genes in CRC. More than 40% of CRCs have p53 mutations, which abrogate its ability to transactivate canonical p53 target genes. Although the remaining CRCs do not have p53 gene mutations, they usually have compromised p53 function due to alterations in genes involved in p53 regulation [16].

In this study, we identified that Ajuba is a novel and negative regulator of p53 by forming a complex with p53 and MDM2 and promoting p53 degradation. Ajuba is overexpressed in most CRC tissues and knock‐down of Ajuba promotes CRC cancer cells apoptosis induced by chemotherapy drugs. Therefore, Ajuba is a potential therapeutic target to treat colorectal cancer.

## 2 Materials and methods

### 2.1 Plasmids

The plasmids of Myc‐Ajuba, Myc‐Ajuba‐LIM, Myc‐Ajuba‐PreLIM, PCDH‐Myc‐Ajuba, PLKO.1‐shAjuba, pGEX‐4T1‐Ajuba and PET28a‐His‐Ajuba have been previously described. The Flag‐p53, HA‐Ub, HA‐MDM2 and Flag‐MDM2 were cloned into pcDNA‐3.1‐vector. The plasmids of p53 transcations were subcloned from pcDNA3.1‐Flag‐p53 plasmids. pGEX‐4T1‐MDM2 and PET28a‐His‐P53 plasmids were subcloned from pCDNA3.1‐HA‐MDM2 and pCDNA3.1‐Flag‐p53 plasmids, respectively.

### 2.2 Tissue samples

Tissue microarray containing 87 pairs of colorectal cancer and paracancerous tissues were purchased from SHANGHAI OUTDO BIOTECH CO., LTD (Shanghai, China) (No. HColA180 Su11). The general condition of the patients and p53 staining of the tissues were attached to the product.

### 2.3 Immunohistochemistry (IHC)

The expression of Ajuba in colorectal cancer and adjacent tissues was detected by IHC using a polyclonal antibody of Ajuba (#4897, CST, dilution 1 : 300). The immunoreactive scoring rules are as previously described. Immunohistochemistry staining scores > 4 points and higher than those in adjacent tissues are considered as high Ajuba expression.

### 2.4 Cell culture, transfection and retroviral infection

The CRC cell lines HCT116 (RRID: CVCL_0291) and RKO (RRID: CVCL_0504) were obtained from National Collection of Authenticated Cell Cultures. All of the cells had the STR authentication. The genomic DNA of the cells was extracted, and the STR loci were amplified by fluorescent primer PCR. Then, the amplification products were subjected to capillary electrophoresis, and the genotyping results of the cells were obtained and compared with the STR database. These cells were cultured in DMEM supplemented with 10% FBS, 2 mm L‐glutamine, and penicillin (50 U·mL^−1^)/streptomycin (50 μg·mL^−1^) at 37 °C under 5% CO_2_ in a humidified chamber. The detailed methods of transfection and infection were previously described [9]. All experiments were performed with mycoplasma‐free cells.

### 2.5 Co‐immunoprecipitation (co‐IP) assay, GST‐pulldown, western blotting and antibodies

Co‐IP, GST‐pulldown and western blotting analysis were performed as described previously. GST‐Ajuba, His‐Ajuba, GST‐MDM2 and His‐p53 protein were expressed and purified in *BL21 E. coli* cells. In p53 degradation assays, cells were incubated with 50 μg·mL^−1^ cycloheximide (CHX) for indicated time and harvested for western blotting, and the p53 degradation was quantitatively analysed using IMAGEJ (National Institutes of Health, Bethesda, MA, USA), and normalized to β‐actin. The antibodies or beads used in these assays were Rabbit anti‐Myc (16286‐1‐AP, Proteintech, Rosemont, PA, USA), Mouse anti‐Myc (13‐2500, Invitrogen, Waltham, MA, USA), Rabbit anti‐Flag (20543‐1‐AP, Proteintech, Rosemont, PA, USA), Rabbit anti‐HA (51064‐2‐AP, Proteintech, Rosemont, PA, USA), mouse anti‐β‐actin (60008‐1‐lg, Proteintech, Rosemont, PA, USA), Rabbit anti‐Ajuba (4897S, Cell Signaling, Danvers, MA, USA), anti‐p53 (sc‐126AC, Santa Cruz, Dallas, TX, USA or 10442‐1‐AP, Proteintech,Rosemont, PA, USA), anti‐MDM2 (sc‐965, Santa cruz), Flag‐M2‐beads (M8823, Sigma, Darmstadt, Germany), anti‐HA beads (88 837, ThermoFisher, Waltham, MA, USA), anti‐Myc beads (B26302, Bimake, Shanghai, China), Protein A/G beads (sc2003, Santacruz, Dallas, TX, USA), Normal mouse IgG (sc‐2025, Santacruz, Dallas, TX, USA) and Normal Rabbit IgG (2729S, Cell Signaling, Danvers, MA, USA). HRP‐conjugated secondary anti‐mouse or anti‐rabbit antibodies were purchased from Proteintech (Rosemont, PA, USA). HRP‐Anti‐mouse light chain secondary antibody was purchased from Jackson ImmunoResearch（West Grove, PA, USA）.

### 2.6 Real‐time RT‐PCR analysis

RNA extraction, reverse transcription and qPCR detailed procedure have been previously described. The primers (5′‐3′) were listed below:

β‐actin: F‐GGACTTCGAGCAAGAGATGG; R‐AGCACTGTGTTGGCGTACAG
p53: F‐CTCCTCAGCATCTTATCCGAGTG; R‐AAACACGCACCTCAAAGCTGTTC
Ajuba: F‐AGGACTACTTCGGCACCTGTATC; R‐ATAGGACTTCCCCATTGCTTGT
Bax: F‐TTTTCTGACGGCAACTTCAACTG; R‐TCTTCTTCCAGATGGTGAGTGAG
P21: F‐GACTTTGTCACCGAGACACCAC; R‐GAGGCACAAGGGTACAAGACAGT

### 2.7 Cell growth and viability assays

The HCT116 or RKO cells were seeded into 96‐well (1000–2000 cells per well) and treated with chemotherapy drugs or DMSO as indicated. The cell growth was detected at indicated times using Cell Counting Kit‐8 (Dojindo Laboratories, Kumamoto Prefecture, Kyushu, Japan). The results were normalized to DMSO control.

### 2.8 AnnexinV‐FITC apoptosis detection

Cells were collected and washed with PBS. Then incubate cell pallet with AnnexinV‐FITC and PI for 5minites, respectively. Analyse AnnexinV‐FITC binding by flow cytometry (Ex = 488 nm, Em = 530 nm).

### 2.9 Statistical analysis

Data shown were repeated at least three independent times, and results are expressed as mean ± SD. *P*‐values were obtained using two‐tailed Student's *t*‐tests.

## 3 Results

### 3.1 Ajuba protein is overexpressed in paired CRC tissues

**Fig. 1:**
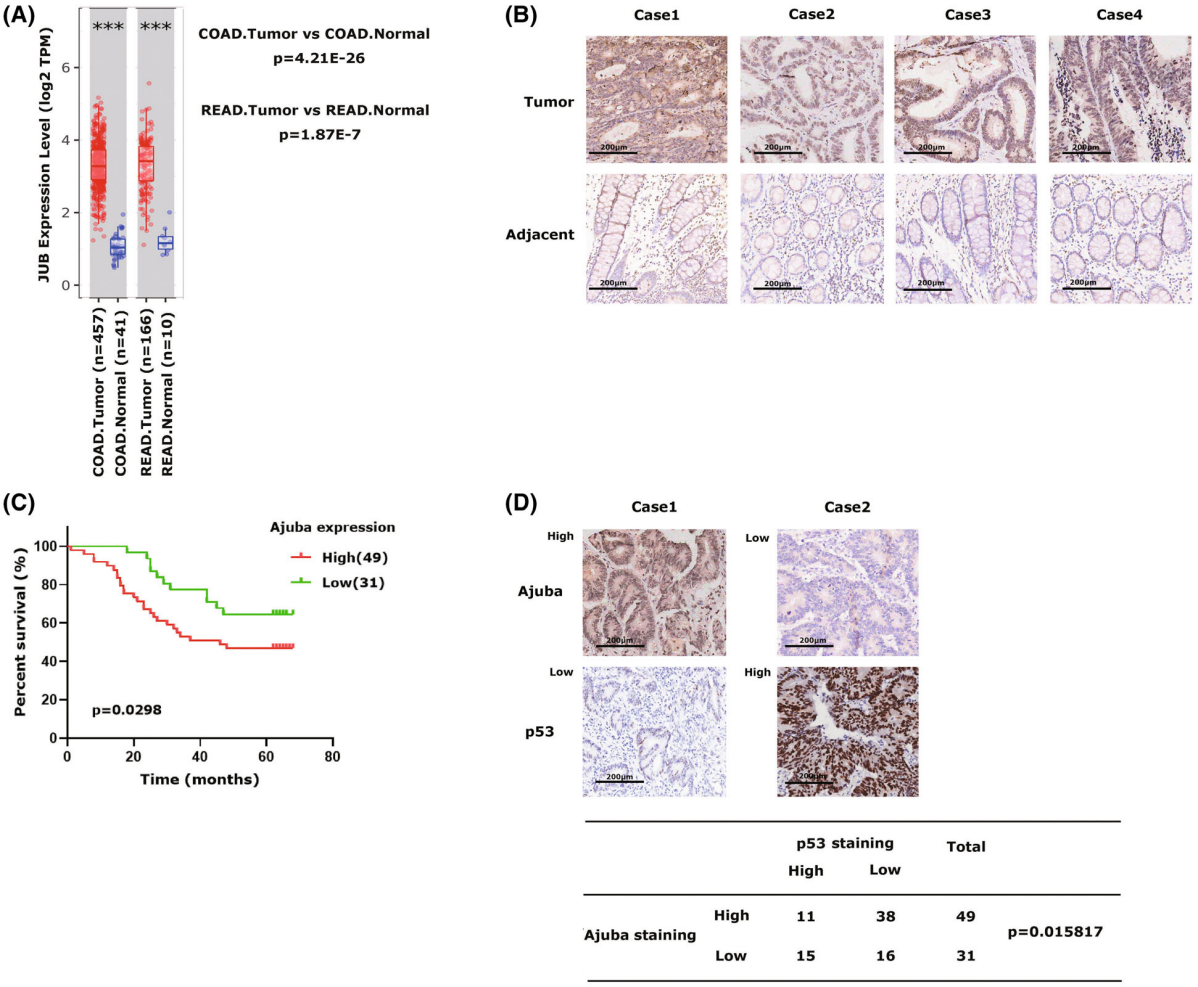
Ajuba protein is overexpressed in paired CRC tissues. (A) The expression of Ajuba in colorectal cancer and normal colorectal tissues was retrieved from the TIMER2.0 online database. The error bars indicate min/max, the *P*‐value was determined by wilcoxon test, ***P < 1E‐4. (B) Representative images of Ajuba immunohistochemical (IHC) staining in colorectal cancer tissues and their paired adjacent normal tissues. Scale bars stands for 200 μm. (C) The correlation analysis between Ajuba protein expression and survival in CRC patients. The P‐value was determined by Gehan–Breslow–wilcoxon test. (D) The table shows the correlation analysis of Ajuba protein expression and p53 protein expression based on IHC results in 80 pairs of colorectal cancer and adjacent colorectal tissues. Statistical analysis was performed with the chi‐square test. Scale bars stands for 200 μm.

At first, we confirmed that Ajuba mRNA expression was indeed significantly higher in colon or rectum adenocarcinoma tissues than normal tissues by exploring the public online database TIMER2.0 (Fig. 1A). By utilizing immunohistochemistry (IHC) method, we analysed the Ajuba protein expression in 80 paired CRC tissues and found that 49 cases had higher Ajuba expression in CRC tissues than in paired‐adjacent nontumour tissues (Fig. 1B) and high expression of Ajuba indicated poorer survival in these CRC patients (Fig. 1C). In addition, we observed that p53 protein expression was always lower in these Ajuba high CRC tissues (Fig. 1D), and this indicated a possible correlation between Ajuba and p53.

### 3.2 Ajuba promotes ubiquitination and degradation of p53

**Fig. 2:**
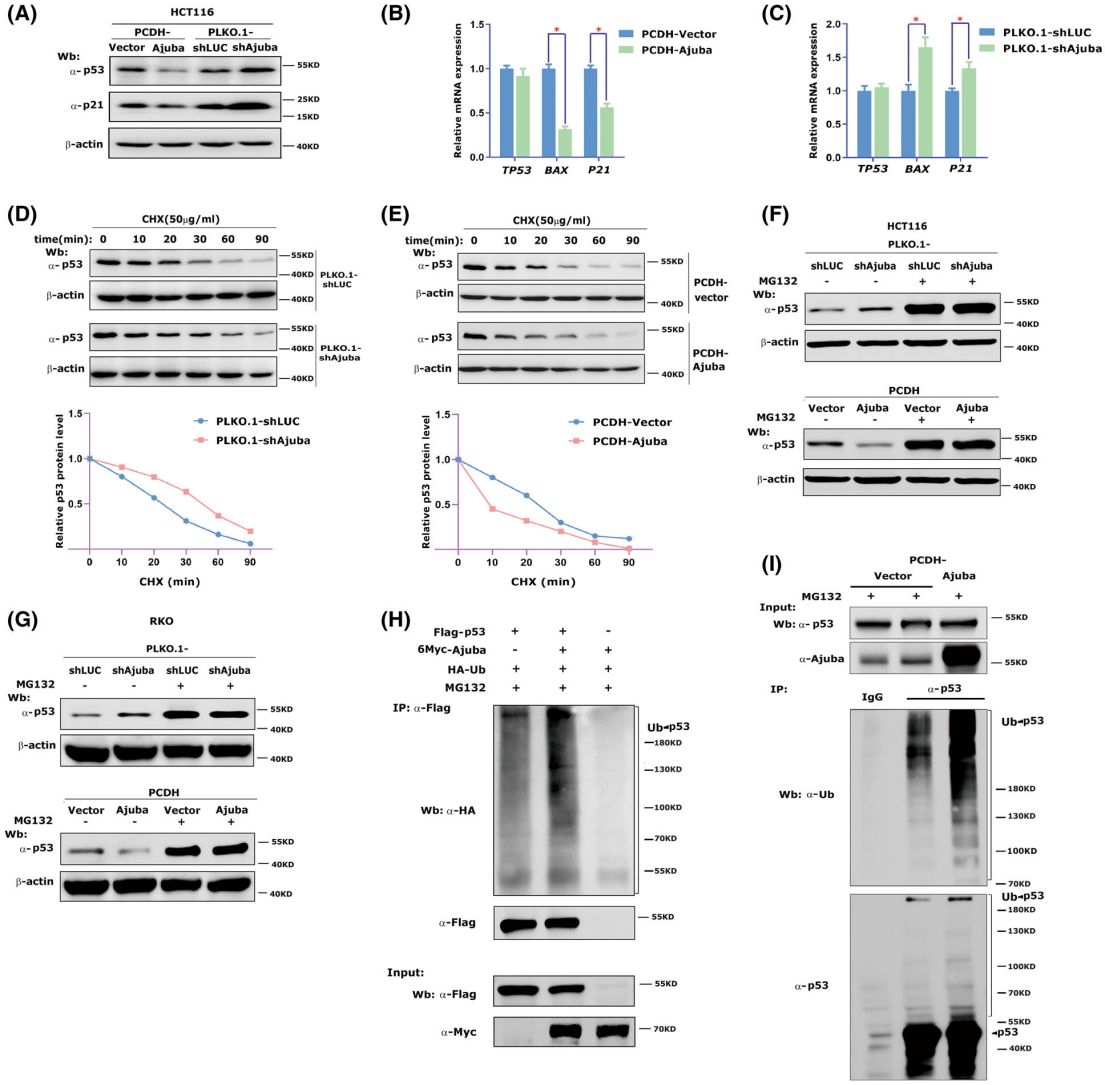
Ajuba promotes ubiquitination and degradation of p53. (A) The protein level of p53 and p21 was detected by western blotting analysis in HCT116 cells with stably overexpressing Ajuba (PCDH‐Ajuba) and control cells (PCDH‐Vector) or HCT116 cells with stably knocking‐down Ajuba (PLKO.1‐shAjuba) or control cells (PLKO.1‐shLUC, shLUC means shLuciferase as control). The experiment has been repeated twice and one representative experiment is shown. (B, C) RT‐qPCR was used to detect the expression of *TP53* and its target genes *P21* and *BAX* in HCT116‐PCDH‐Ajuba or PCDH‐vector cells (B) and HCT116‐PLKO.1‐shAjuba or PLKO.1‐shLuc cells (C) (data were shown as mean ± SD, three independent times, *P*‐values were determined by two‐tailed *t*‐test, *P < 0.05). (D, E) CHX (50 μg·mL^−1^) was added into HCT116‐PLKO.1‐shAjuba or PLKO.1‐shLuc cells (D) or HCT116‐PCDH‐Ajuba or PCDH‐vector cells (E) for different time and the protein level of p53 was detected by western blotting analysis. The experiment has been repeated more than three times (D) and twice (E), one representative experiment is shown. (F, G) MG132 (10 μm) was added to HCT116 cells (F) or RKO cells (G) with stable Ajuba overexpression or shAjuba for 6 h and p53 protein expression was detected in western blotting. The experiment has been repeated twice and one representative experiment is shown. (H) HCT116 cells were transfected with Flag‐p53, Myc‐Ajuba and HA‐Ub and harvested after MG132 treatment (10 μm, 4–6 h). Flag antibody was used to enrich Flag‐p53, and HA antibody was used to detect ubiquitinated p53 protein. A single experiment was performed. (I) HCT116‐PCDH‐Ajuba or PCDH‐vector cells were treated with MG132 (10 μm, 4–6 h) and the endogenous p53 protein was enriched by using p53 antibody. Ub antibody was used to detect the ubiquitinated p53 protein. The experiment has been repeated twice and one representative experiment is shown.

To test whether Ajuba plays a role in regulating p53 expression, we established stably overexpressing Ajuba or shAjuba in HCT116 or RKO cell lines (Fig. S1A–D). In HCT116 cells, we found that Ajuba overexpression decreased the protein levels of p53 and its downstream regulatory protein p21 and conversely shAjuba increased their expression (Fig. 2A). At mRNA level, we observed that Ajuba overexpression decreased p53 target genes *P21* and *Bax* expression and vice versa (Fig. 2B,C). However, there was no significant difference in *TP53* RNA levels in overexpressing or shAjuba cells (Fig. 2B,C). This indicates that Ajuba regulates p53 expression at post‐transcriptional level.

It is well known that p53 expression is primarily controlled by protein stability and ubiquitination‐proteosome‐mediated degradation. First, we utilized cycloheximide (CHX), a protein synthesis inhibitor, to test whether Ajuba could influence p53 protein stability in HCT116 or RKO cells. The results showed that shAjuba increased p53 stability (Fig. 2D and Fig. S2) and conversely overexpressing Ajuba decreased p53 stability (Fig. 2E). In HCT116 or RKO cells, we found that the effect of Ajuba on p53 protein expression was significantly blocked after the proteosome inhibitor MG132 treatment (Fig. 2F,G). In addition, Ajuba overexpression indeed enhanced the ubiquitination level of p53 (Fig. 2H,I). These results indicate that Ajuba regulates p53 protein by promoting the ubiquitination and degradation of p53.

### 3.3 Ajuba decreases chemotherapy drugs‐induced apoptosis and cell growth inhibition

**Fig. 3:**
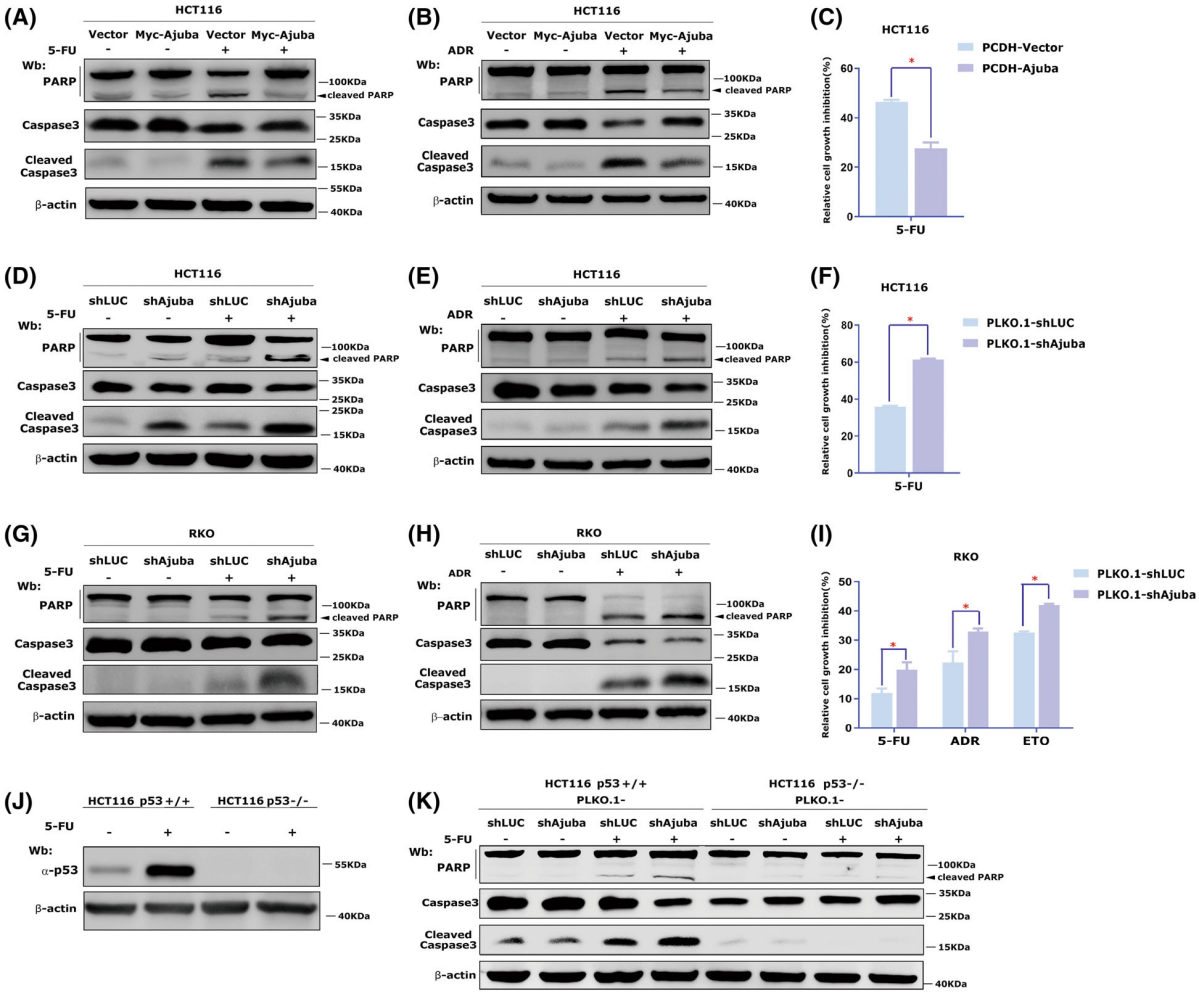
Ajuba decreases chemotherapy drugs‐induced apoptosis and cell growth inhibition. (A, B) HCT116‐PCDH‐Ajuba or PCDH‐vector cells were treated with 50 μm 5‐FU (A) or 0.5 μm Adriamycin (ADR) (B) for 48 h, cleaved‐PARP and cleaved‐caspase3 were detected by western blotting.The experiment has been repeated three times and one representative experiment is shown. (C) HCT116‐PCDH‐Ajuba or PCDH‐vector cells were treated with 50 μm 5‐FU for 48 h, and cell viability was examined by CCK8 assay (data were shown as mean ± SD, three independent times, *P*‐values were determined by two‐tailed *t*‐test, *P < 0.05). (D, E) HCT116‐PLKO.1‐shAjuba or PLKO.1‐shLUC cells were treated with 50 μm 5‐FU (D) or 0.5 μm Adriamycin (ADR) (E) for 48 h, cleaved‐PARP and cleaved‐caspase3 were detected by western blotting. The experiment has been repeated three times and one representative experiment is shown. (F) HCT116‐PLKO.1‐shAjuba or PLKO.1‐shLUC cells were treated with 50 μm 5‐FU for 48 h, cell viability was examined by CCK8 assay (data was shown as mean ± SD, three independent times, *P*‐values were determined by two‐tailed *t*‐test, *P < 0.05). (G, H) Stably transfected shAjuba RKO cells (RKO‐PLKO.1‐shAjuba) or control cells (PLKO.1‐shLUC) were treated with 50 μm 5‐FU (G) or 0.5 μm Adriamycin (ADR) (H) for 48 h, cleaved‐PARP and cleaved‐caspase3 were detected by western blotting. The experiment has been repeated three times and one representative experiment is shown. (I) RKO‐PLKO.1‐shAjuba or PLKO.1‐shLUC cells were treated with 50 μm 5‐FU, 50 μm Etoposide (ETO) or 0.5 μm Adriamycin (ADR) for 48 h, cell viability was examined by CCK8 assay (data were shown as mean ± SD, three independent times, *P*‐values were determined by two‐tailed *t*‐test, *P < 0.05). (J) P53 expression in HCT116 p53^+/+^ and HCT116 p53^−/−^ cells was detected after 5‐FU treatment (25 μm, 24 h) by using western blotting. The experiment has been repeated twice and one representative experiment is shown. (K) HCT116 p53^+/+^ or HCT116 p53^−/−^ cells with stable shAjuba or shLUC were treated with 50 μm 5‐FU for 48 h, cleaved‐PARP and cleaved‐caspase3 were detected by western blotting.The experiment has been repeated three times and one representative experiment is shown.

P53 is critical for chemotherapeutic drug induced apoptosis in CRC. Thus, we speculated that Ajuba might inhibit chemotherapeutic drug‐mediated apoptosis in CRC cells by regulating p53. In HCT116 cells, 5‐FU or Adriamycin (ADR) treatment significantly induced cleaved‐PARP and cleaved‐caspase‐3 level that indicated cell apoptosis induction (Fig. 3A,B). However, overexpressing Ajuba significantly decreased the level of cleaved‐PARP and cleaved‐caspase‐3 induced by these chemotherapy drugs (Fig. 3A,B). In addition, Ajuba overexpression also significantly decreased 5‐FU‐induced cell growth inhibition (Fig. 3C). Conversely, shAjuba enhanced 5‐FU, Adriamycin (ADR) or Oxaliplatin (Oxa)‐induced cell apoptosis and cell growth inhibition (Fig. 3D–F and Fig. S3A). In RKO cells, similar effect of Ajuba overexpression or knocking‐down on 5‐FU or Adriamycin (ADR) or Etoposide (ETO)‐induced apoptosis and cell growth inhibition was observed (Fig. 3G–I and Fig. S3B,C). In addition, in HCT116 p53^−/−^ cells, shAjuba and/or 5‐FU treatment failed to induce PARP or caspase‐3 cleavage (Fig. 3J,K), which indicates the effect of Ajuba on apoptosis is largely dependent on p53.

### 3.4 Ajuba directly interacts with p53

**Fig. 4:**
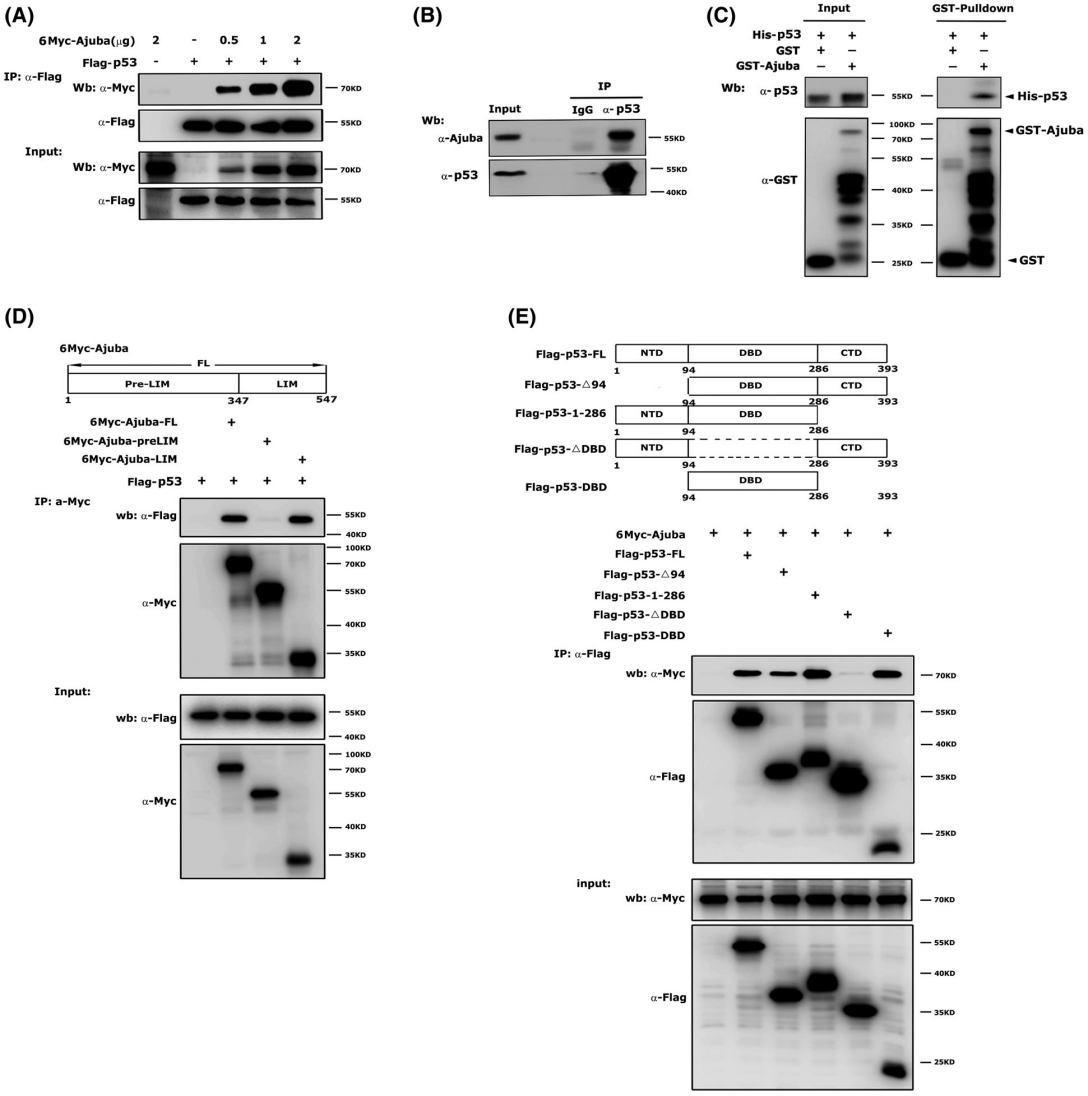
Ajuba directly interacts with p53. (A) Plasmids encoding Myc‐Ajuba and Flag‐p53 were transfected into HCT116 cells, and transfected cells were incubated with MG132 (10 μm) for 4–6 h before being harvested for co‐IP assay by using Flag‐M2‐beads. The experiment has been repeated three times and one representative experiment is shown. (B) HCT116 cells were incubated with MG132 (10 μm) for 4–6 h before being harvested for endogenous co‐IP assay by using p53 antibody or normal control IgG.A single experiment was performed. (C) GST‐Ajuba and His‐p53 expressed and purified from *BL21‐E. coli* strains were used to perform *in vitro* GST‐pulldown assay and p53 antibody or GST antibody was used to detect indicated protein. A single experiment was performed. (D) Full length (FL) or truncations of Ajuba expressing plasmids and Flag‐p53 plasmids were transfected into HCT116 cells, and the transfected cells were incubated with MG132 (10 μm) for 4–6 h before being harvested for co‐IP assay by using Myc antibody. A single experiment was performed. (E) Full length (FL) or truncations of p53 expressing plasmids and Myc‐Ajuba plasmids were transfected into HCT116 cells, and the transfected cells were incubated with MG132 (10 μm) for 4–6 h before being harvested for co‐IP assay by using Flag‐M2 beads. CTD, C‐terminal domain; DBD, DNA binding domain; NTD, N‐terminal domain. A single experiment was performed.

Ajuba functions as a scaffold protein by mediating protein–protein interaction. First, we wanted to test whether Ajuba could interact with p53. We transfected Ajuba and p53 plasmids into HCT116 cells and carried out co‐immunoprecipitation (co‐IP) assays. The results clearly showed that Flag‐p53 could pull down Myc‐Ajuba in a dose‐dependent manner (Fig. 4A). Endogenous co‐IP assays in HCT116 cells also confirmed their interaction (Fig. 4B). To figure out whether their interaction was direct, we expressed and purified GST‐Ajuba or His‐p53 proteins by using *BL21*‐*E. coli* cells and *in vitro* GST‐pull down assay was performed. The results showed GST‐Ajuba could pull down His‐p53 protein *in vitro* (Fig. 4C), which suggests that Ajuba directly interacts with p53.

Ajuba is structurally characterized by a N‐terminal Pre‐LIM domain and a C‐terminal LIM domains. To further explore which segment or domain specifically mediates the interaction between Ajuba and p53, we constructed various plasmids expressing different segments or domains of Ajuba or p53 for co‐IP assays. The results showed that Ajuba interacted with the DNA‐binding domain (DBD) of p53 through its C‐terminal LIM domain (Fig. 4D,E).

### 3.5 Ajuba directly interacts with MDM2

**Fig. 5:**
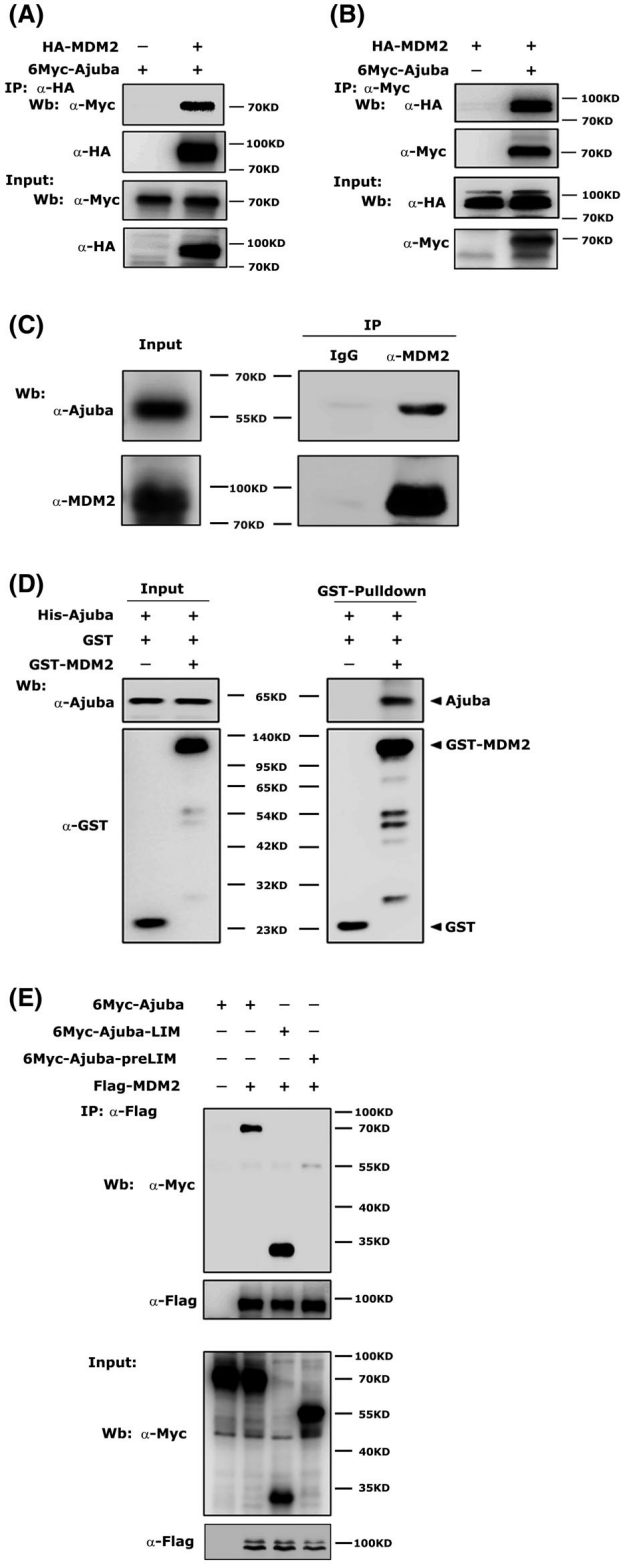
Ajuba directly interacts with MDM2. (A, B) Plasmids encoding Myc‐Ajuba or HA‐MDM2 were transfected into HCT116 cells, and co‐IP assay was performed by using HA antibody (A) or Myc antibody (B). The experiment has been repeated three times and one representative experiment is shown. (C) HCT116 cells were harvested for co‐IP assay by using MDM2 antibody or normal control IgG. A single experiment was performed. (D) GST‐MDM2 and His‐Ajuba expressed and purified in *BL21*‐*E. coli* strains were used for *in vitro* GST‐pulldown assay and Ajuba antibody or GST antibody was used to detect indicated protein. A single experiment was performed. (E) Full length (FL) or truncations of Ajuba expressing plasmids and Flag‐MDM2 plasmids were transfected into HCT116 cells, and co‐IP assay was performed by using Flag‐M2‐beads. A single experiment was performed.

MDM2 is the most critical E3 ligase mediating p53 ubiquitination and degradation. We speculated that Ajuba might also interact with MDM2. Indeed, Co‐IP assay results showed HA‐MDM2 and Myc‐Ajuba could pull down each other (Fig. 5A,B). Endogenous co‐IP result in HCT116 cells also confirmed the interaction between Ajuba and MDM2 (Fig. 5C). *In vitro* GST‐pull down assay revealed GST‐MDM2 could pulldown His‐Ajuba protein that indicated the interaction between MDM2 and Ajuba was direct (Fig. 5D). In addition, we also reveal that Ajuba interacts with MDM2 through its C‐terminal LIM domain (Fig. 5E).

### 3.6 Ajuba forms a complex with MDM2/p53 and promotes MDM2‐mediated p53 ubiquitination

**Fig. 6:**
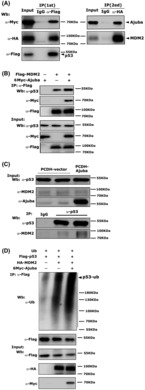
Ajuba enhances MDM2/p53 interaction and promotes MDM2‐mediated p53 ubiquitination. (A) Plasmids encoding Myc‐Ajuba, HA‐MDM2 and Flag‐p53 were co‐transfected into HCT116 cells and the transfected cells were incubated with MG132 (10 μm) for 4–6 h before being harvested for sequential co‐IP assays. The first round co‐IP assay was carried out by using Flag antibody or normal IgG and 3XFlag‐peptides were used to elute the enriched protein which was subjected to the second round co‐IP assay by using HA antibody or normal IgG. A single experiment was performed. (B) Flag‐MDM2 and Myc‐Ajuba plasmids were transfected into HCT116 cells, and the transfected cells were incubated with MG132 (10 μm) for 4–6 h before being harvested for co‐IP assay by using Flag‐M2‐beads. Endogenous p53 was detected by using p53 antibody. The experiment has been repeated twice and one representative experiment is shown. (C) HCT116‐PCDH‐Ajuba or PCDH‐vector cells were incubated with MG132 (10 μm) for 4–6 h before being harvested for endogenous co‐IP assay by using p53 antibody or normal control IgG. The experiment has been repeated twice and one representative experiment is shown. (D) Plasmids encoding Myc‐Ajuba, HA‐MDM2 or Flag‐p53 were transfected into HCT116 cells as indicated and p53 protein was enriched by using Flag‐M2‐beads. The ubiquitination level of p53 was detected by Ub antibody. The experiment has been repeated three times and one representative experiment is shown.

The direct interaction of Ajuba with p53 and MDM2 lead us to test whether Ajuba could form a complex with p53/MDM2 by using sequential co‐IP assay. We co‐expressed Flag‐p53, Myc‐Ajuba and HA‐MDM2 in HCT116 cells. The first round co‐IP assay was performed with the Flag antibody to enrich the proteins that interacted with p53 protein. The enriched protein was eluted by using 3XFlag peptides. The eluted protein was also used to conduct the second round co‐IP assay with the HA antibody to pull down the proteins that interacted with MDM2. The first round co‐IP result showed both Myc‐Ajuba and HA‐MDM2 were pulled down by Flag‐p53 and Myc‐Ajuba was indeed pulled down by HA‐MDM2 in the sequential second round co‐IP (Fig. 6A). Therefore, the sequential co‐IP assay indicates that Ajuba, MDM2 and P53 form a complex. To test whether Ajuba had any influence on the association between p53 and MDM2, we transfected p53, MDM2 or Ajuba expressing plasmids into HCT116 cells, and co‐IP results showed Ajuba could increase the interaction between p53 and MDM2 (Fig. 6B). In addition, we also observed the endogenous p53/MDM2 association could be enhanced by Ajuba overexpression in HCT116 cells (Fig. 6C). Conversely, shAjuba evidently decreased the interaction between p53 and MDM2 in RKO cells (Fig. S4). Finally, in HCT116 cells, we found Ajuba overexpression enhanced MDM2‐mediated p53 ubiquitination (Fig. 6D).

### 3.7 Ajuba expression can be induced by chemotherapy drugs

**Fig. 7:**
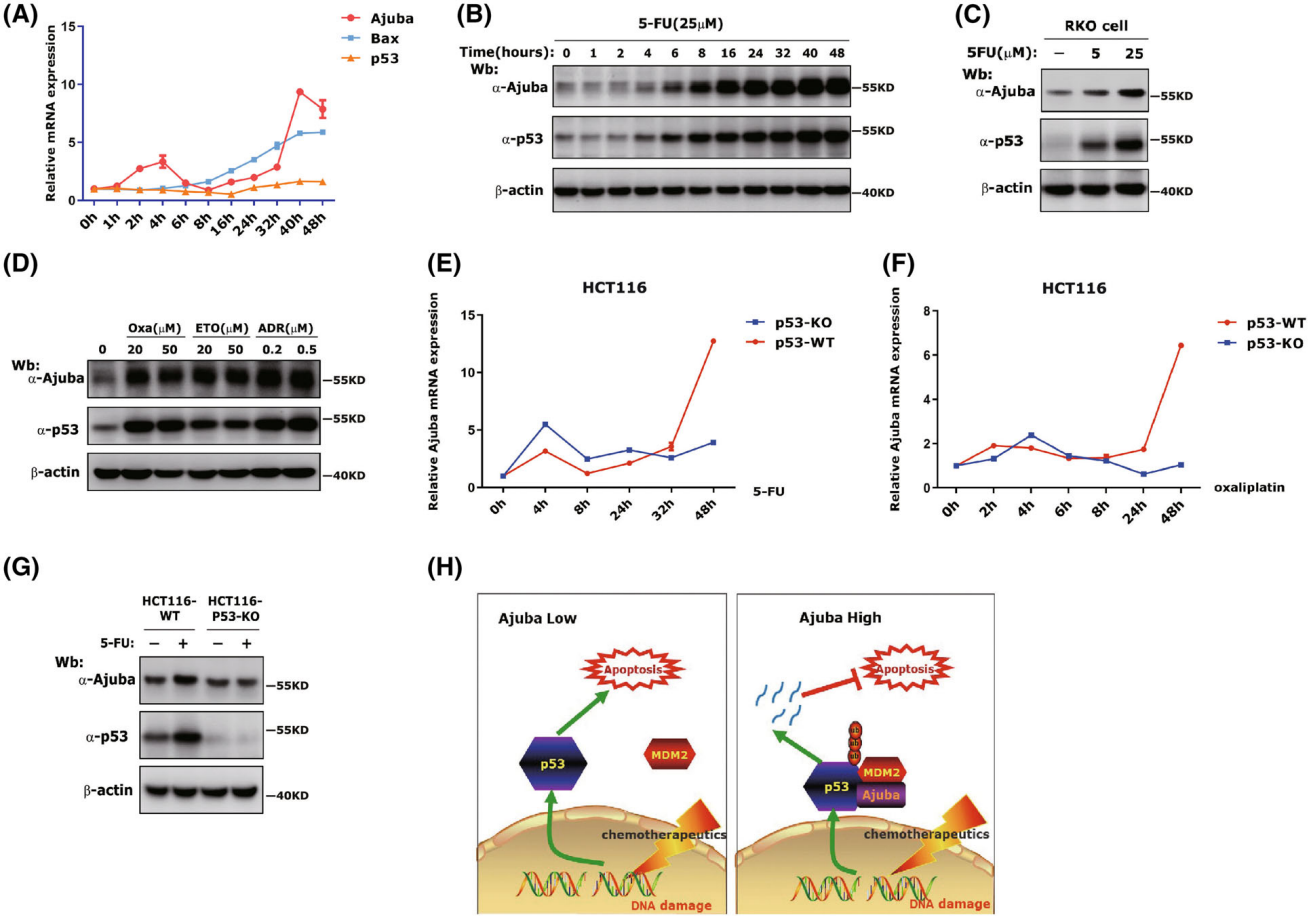
Ajuba expression can be induced by chemotherapy drugs. (A) HCT116 cells were incubated with 25 μm 5‐FU for indicated time. The expression of p53, Bax and Ajuba was detected by qRT‐PCR (data was shown as mean ± SD, three independent times). (B) HCT116 cells were incubated with 25 μm 5‐FU for indicated time. The expression of p53 and Ajuba protein was detected by western blotting.The experiment has been repeated three times and one representative experiment is shown. (C) RKO cells were incubated with 5 or 25 μm 5‐FU for 48 h and Ajuba expression was detected by western blotting. The experiment has been repeated twice and one representative experiment is shown. (D) HCT116 cells were incubated with Oxaliplatin (Oxa), Adriamycin (ADR) or Etoposide (ETO) for 48 h and Ajuba expression was detected by western blotting. The experiment has been repeated twice and one representative experiment is shown. (E, F) HCT116 p53^+/+^ or HCT116 p53^−/−^ cells were incubated with 25 μm 5‐FU (E) or Oxaliplatin (Oxa) (F) for indicated time and the expression of Ajuba was detected by qRT‐PCR (data was shown as mean ± SD, three independent times). (G) HCT116 p53^+/+^ or HCT116 p53^−/−^ cells were incubated with 25 μm 5‐FU for 48 h, and the expression of Ajuba was detected by western blotting. The experiment has been repeated twice and one representative experiment is shown. (H) Ajuba is a novel negative regulator of p53. When Ajuba expression is low, chemotherapy drugs induce p53 stabilization and cell apoptosis. When Ajuba expression is high, it forms a complex with p53/MDM2 and enhances p53/MDM2 interaction and promotes p53 degradation and suppresses chemotherapy drug‐induced cell apoptosis.

To explore whether Ajuba expression was changed in chemotherapy drugs treatment, we utilized 5‐FU to treat HCT116 cells for different time course. The RT‐PCR results showed that the p53 expression was almost stable or slightly changed and the expression of p53 target gene *Bax* was gradually induced with the duration of drug treatment (Fig. 7A). Surprisingly, we also observed Ajuba expression was significantly induced by 5‐FU treatment. Moreover, Ajuba expression was moderately induced at early stage of 5‐FU treatment (2–6 h) and then declined and finally more significantly induced again at late stage of 5‐FU treatment (16–48 h; Fig. 7A). We also found Ajuba protein expression was significantly induced after 5‐FU treatment (Fig. 7B). In RKO cells, we found Ajuba protein was clearly induced by 5‐FU in dose‐dependent manner (Fig. 7C). Other chemotherapy drugs including Oxaliplatin (Oxa), Adriamycin (ADR) or Etoposide (ETO) also evidently induced Ajuba protein expression in HCT116 cells (Fig. 7D).

To explore whether Ajuba expression induced by chemotherapy drugs was dependent on p53, we test the induction of Ajuba expression in HCT116 p53^−/−^cells. The results showed that, although the moderate early‐stage induction of Ajuba mRNA was almost not influenced by the loss of p53, the striking late‐stage induction of Ajuba mRNA was almost disappeared in the absence of p53 (Fig. 7E,F and Fig. S5). In addition, we also found Ajuba protein induction by 48‐h 5‐FU treatment was largely suppressed in HCT116 p53^−/−^cells (Fig. 7G).

Overall, our studies indicate Ajuba is a novel negative regulator of p53. Ajuba directly interacts with p53 and MDM2 and promotes p53 degradation. High expression of Ajuba in CRC cancer can inhibit chemotherapy drugs‐induced apoptosis and mediate chemotherapy resistance and Ajuba may be an important therapeutic target for CRC cancer treatment (Fig. 7H).

## 4 Discussion

Chemotherapy drugs are commonly used to trigger apoptotic signals via inducing DNA damage response that is largely dependent on p53. Many proteins have been found to regulate p53 protein expression or function to influence the effect of chemotherapy [17]. Ajuba was demonstrated to have important roles in various tumours, such as breast cancer [9], cervical cancer [21], head and neck squamous cell carcinoma [17, 22] and CRC [14, 23]. In CRC cells, the detailed function of Ajuba is not very clear. In this study, we found Ajuba directly interacts with p53 and MDM2 and promotes p53 ubiquitination and degradation and hence inhibits chemotherapy drug‐induced apoptosis.

In 80 pairs of CRC and adjacent nontumour tissues, we found Ajuba protein expression was increased in CRC tissues compared with adjacent nontumour tissues in more than 60% cases (49 of 80) that indicates its potential oncogenic function in CRC cells. Several reports have discovered that Ajuba could promote CRC survival, migration and metastasis [14, 23, 24]. In addition, Ajuba was proven to promote tumour cell proliferation such as in breast cancer and pancreatic cancer [9, 25]. In CRC cells, we found Ajuba overexpression suppressed apoptosis induced by 5‐FU, Oxaliplatin and other chemotherapy drugs. Conversely, knocking‐down Ajuba enhanced apoptosis induced by these chemotherapy drugs. The regulation of Ajuba on apoptosis is largely dependent on p53, because in the absence of p53, knocking‐down Ajuba was unable to promote apoptosis induced by chemotherapy drugs. These results indicate high Ajuba expression in CRC may lead to chemotherapy resistance. In CRC cells, apoptosis trigger by chemotherapy drugs such as 5‐FU is largely dependent on p53 [18, 19]. Interestingly, we found Ajuba overexpression or knocking‐down decreased or increased p53 stability, respectively.

Ajuba can shuttle between nucleus and cytoplasm, and it functions as a scaffold protein that mediate the assembly of different protein complexes and participate in the regulation of various signalling pathways [5]. In nucleus, Ajuba interacts with Snail and PRMT5, it enhances Snail/PRMT5 association and functions as Snail co‐repressor in this protein complex [8]. Ajuba also interacts with CBP/P300 or DBC1 and forms multiprotein complex with nuclear receptors, such as ERa and PPARγ, LXRa [6, 9, 26]. In cytoplasm, Ajuba mediates various protein–protein interactions and regulates many signalling pathways including Hippo, Wnt and Notch, et al. [5, 11]. Many proteins have been reported to regulate p53/MDM2 interaction and influence apoptosis, such as PACT [27], YY1 [28, 29] and Ribosomal protein S7 [30, 31]. In this study, we revealed for the first time that Ajuba directly interacts with p53 and MDM2 and forms protein complex with p53/MDM2 and also promotes p53 ubiquitination. Ajuba protein contains three tandem LIM domains (LIM1/2/3) which is the primary domain mediating interaction of Ajuba with other proteins, such as CBP/P300, Snail, Aurora‐A [7, 9, 32]. Here, we found Ajuba interacted with p53 and MDM2 through its LIM domains. Further research is needed to identify which LIM domain of Ajuba, respectively, mediating its association with MDM2 and p53.

Many p53 regulators influence p53 stability or function in a negative or positive feedback manner, such as MDM2 [33], COP1 [34], P14ARF [35, 36], TRIM32 [37] and TRIM67 [38]. Here, we found Ajuba expression can be significantly induced by chemotherapy drugs. Moreover, Ajuba transcription can be induced in early or late stage of chemotherapy drugs treatment. Notably, Ajuba transcription was moderately induced at early stage (2–6 h after chemotherapy drugs treatment) and more strikingly induced at late stage (16–48 h after chemotherapy drugs treatment). However, Ajuba protein expression was gradually induced and then maintained at high level in the chemotherapy treatment period. It probably because Ajuba protein is a relatively stable protein in cells [39]. In addition, we found that the transcriptional induction of Ajuba is not dependent on p53 at the early stage of chemotherapy drugs treatment, because in HCT116 p53^−/−^cells, Ajuba transcription was still induced by chemotherapy drugs. However, at late stage of chemotherapy drugs treatment, the transcriptional induction of Ajuba was almost disappeared in HCT116 p53^−/−^cells, which indicated late‐stage Ajuba expression induction was largely dependent on p53. Further research is needed to identify the real molecular mechanism of Ajuba expression induction and clarify whether Ajuba is a direct target gene of p53 particularly at the late stage of chemotherapy treatment.

## 5 Conclusions

Overall, Ajuba is a novel negative regulator of p53 and inhibits apoptosis induced by chemotherapy drugs in a negative feedback manner. Ajuba directly interacts with p53/MDM2 and enhances p53/MDM2 interaction and promotes p53 degradation. These results indicate high expression of Ajuba in CRC can mediate chemotherapy resistance, and Ajuba may be a potential therapeutic target for colorectal cancer treatment.

## Conflict of interest

The authors declare that they have no known competing financial interests or personal relationships that could have appeared to influence the work reported in this paper.

## Author contributions

FC and JZ were the project supervisors. QL performed part of the data analysis. BX performed the experiments, analysed the data and wrote the manuscript.

### Peer review

The peer review history for this article is available at https://www.webofscience.com/api/gateway/wos/peer‐review/10.1002/1878‐0261.13421.

## Supporting information


**Fig. S1.** Ajuba expression in Ajuba stably overexpressing or shAjuba cells. A‐B: The expression of Ajuba in Ajuba stably overexpressing (PCDH‐Ajuba) HCT116 cells and control cell PCDH‐Vector (A) or Ajuba stably knock‐down (PLKO.1‐shAjuba) HCT116 cells and control cell PLKO.1‐shLUC (shLUC means shLuciferase as control) (B) was detected by western blotting. C‐D: The expression of Ajuba in RKO‐PCDH‐Ajuba/Vector (C) or RKO‐PLKO.1‐shAJUBA/shLUC (D) cells was detected by western blotting.

**Fig. S2.** shAjuba enhanced p53 stability in RKO cells. Ajuba knock‐down RKO cells (RKO‐PLKO.1‐shAjuba) and control cells (RKO‐PLKO.1‐shLUC) were incubated with CHX (50 μg/mL) for indicated times. The protein level of p53 was detected by western blotting.

**Fig. S3.** Ajuba inhibited apoptosis induced by chemotherapy drugs. A: HCT116‐PLKO.1‐shAjuba or PLKO.1‐shLUC cells were treated with 50 μM Oxaliplatin for 48 h，apoptosis was detected by Annexin V‐FITC/PI apoptotic analysis (data was shown as mean ± sd, three independent times, *P < 0.05). B: RKO‐PCDH‐Ajuba or RKO‐PCDH‐vector cells were treated with 50 μM 5‐FU for 48 h and cleaved‐PARP and cleaved‐caspase3 were detected by western blotting. C: RKO‐PLKO.1‐shAjuba or RKO‐PLKO.1‐shLUC cells were treated with 50 μM Etoposide (ETO) for 48 h, cleaved‐PARP and cleaved‐caspase3 were detected by western blotting.

**Fig. S4.** shAjuba inhibited the interaction between p53 and MDM2. RKO‐PLKO.1‐shAjuba and RKO‐PLKO.1‐shLUC cells were incubated with MG132 (10 μM) for 4–6 h before being harvested for endogenous co‐IP assay by using p53 antibody or normal control IgG.

**Fig. S5.** Ajuba expression can be induced by chemotherapy drugs. HCT116 p53^+/+^ or HCT116 p53^−/−^ cells were incubated with 0.2μΜ Adriamycin (ADR) for indicated time and the RNA‐expression of Ajuba was detected by RT‐qPCR (data were shown as mean ± sd, three independent times).

## Data Availability

The data that support the findings of this study are available from the corresponding author [chenfx@sjtu.edu.cn] upon reasonable request.
