# Supplementary figures and images for: LIM domain‐containing protein Ajuba inhibits chemotherapy‐induced apoptosis by negatively regulating p53 stability in colorectal cancer cells
Source: Mol Oncol. 2023 Apr 3;17(8):1678–91. doi: 10.1002/1878-0261.13421 (PMC10399714; doi:10.1002/1878-0261.13421)

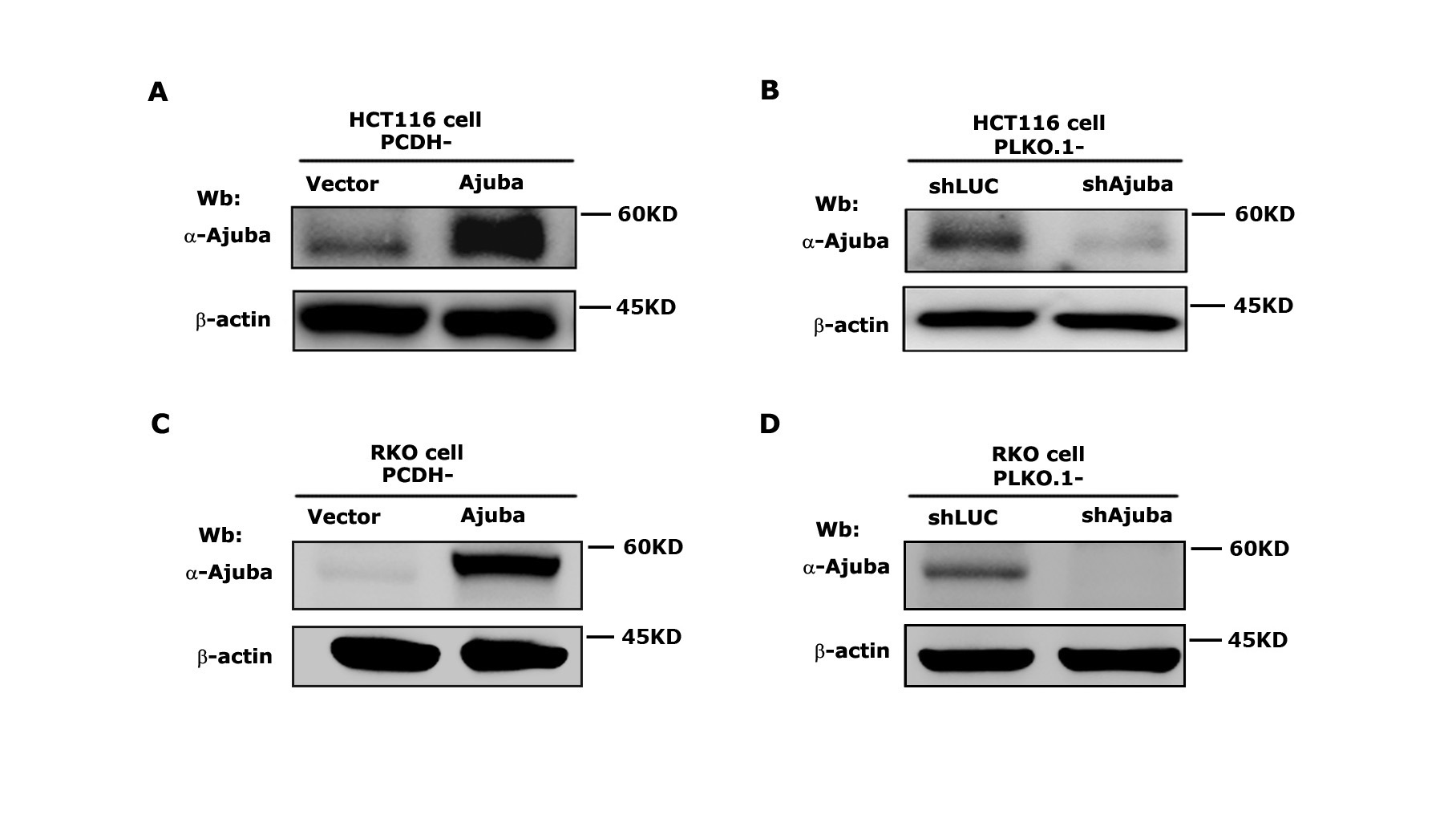

Supplement: Supplementary file 1 — Fig. S1. Ajuba expression in Ajuba stably overexpressing or shAjuba cells. A‐B: The expression of Ajuba in Ajuba stably overexpressing (PCDH‐Ajuba) HCT116 cells and control cell PCDH‐Vector (A) or Ajuba stably knock‐down (PLKO.1‐shAjuba) HCT116 cells and control cell PLKO.1‐shLUC (shLUC means shLuciferase as control) (B) was detected by western blotting. C‐D: The expression of Ajuba in RKO‐PCDH‐Ajuba/Vector (C) or RKO‐PLKO.1‐shAJUBA/shLUC (D) cells was detected by western blotting. [file MOL2-17-1678-s002.jpg]

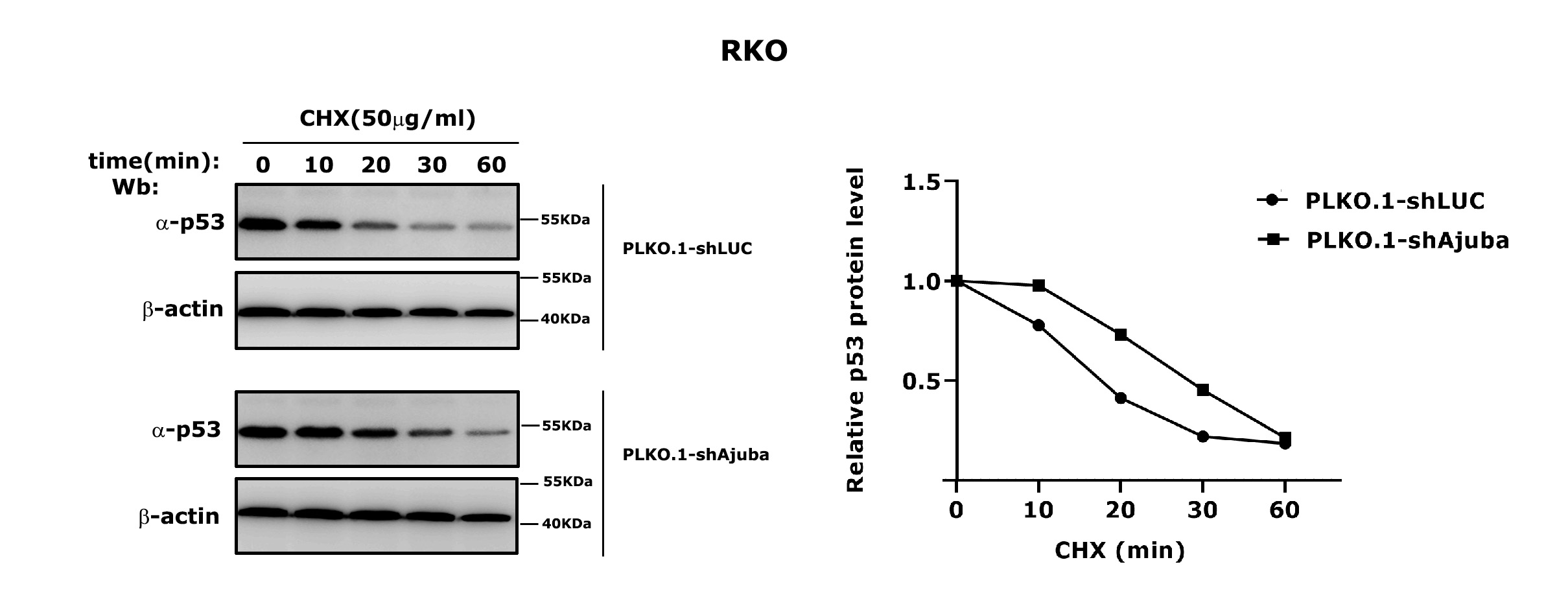

Supplement: Supplementary file 2 — Fig. S2. shAjuba enhanced p53 stability in RKO cells. Ajuba knock‐down RKO cells (RKO‐PLKO.1‐shAjuba) and control cells (RKO‐PLKO.1‐shLUC) were incubated with CHX (50 μg/mL) for indicated times. The protein level of p53 was detected by western blotting. [file MOL2-17-1678-s003.jpg]

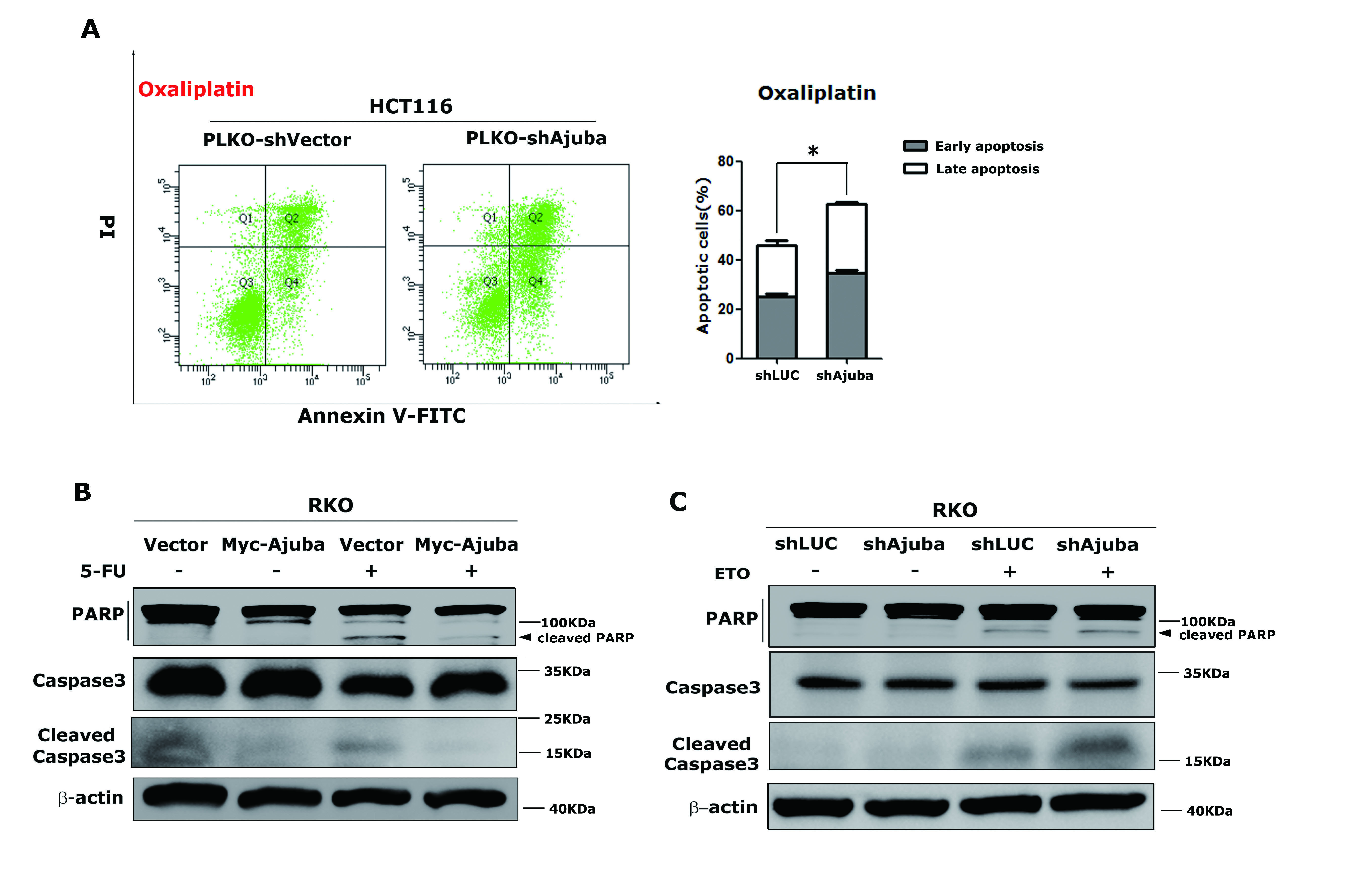

Supplement: Supplementary file 3 — Fig. S3. Ajuba inhibited apoptosis induced by chemotherapy drugs. A: HCT116‐PLKO.1‐shAjuba or PLKO.1‐shLUC cells were treated with 50 μM Oxaliplatin for 48 h，apoptosis was detected by Annexin V‐FITC/PI apoptotic analysis (data was shown as mean ± sd, three independent times, *P < 0.05). B: RKO‐PCDH‐Ajuba or RKO‐PCDH‐vector cells were treated with 50 μM 5‐FU for 48 h and cleaved‐PARP and cleaved‐caspase3 were detected by western blotting. C: RKO‐PLKO.1‐shAjuba or RKO‐PLKO.1‐shLUC cells were treated with 50 μM Etoposide (ETO) for 48 h, cleaved‐PARP and cleaved‐caspase3 were detected by western blotting. [file MOL2-17-1678-s001.jpg]

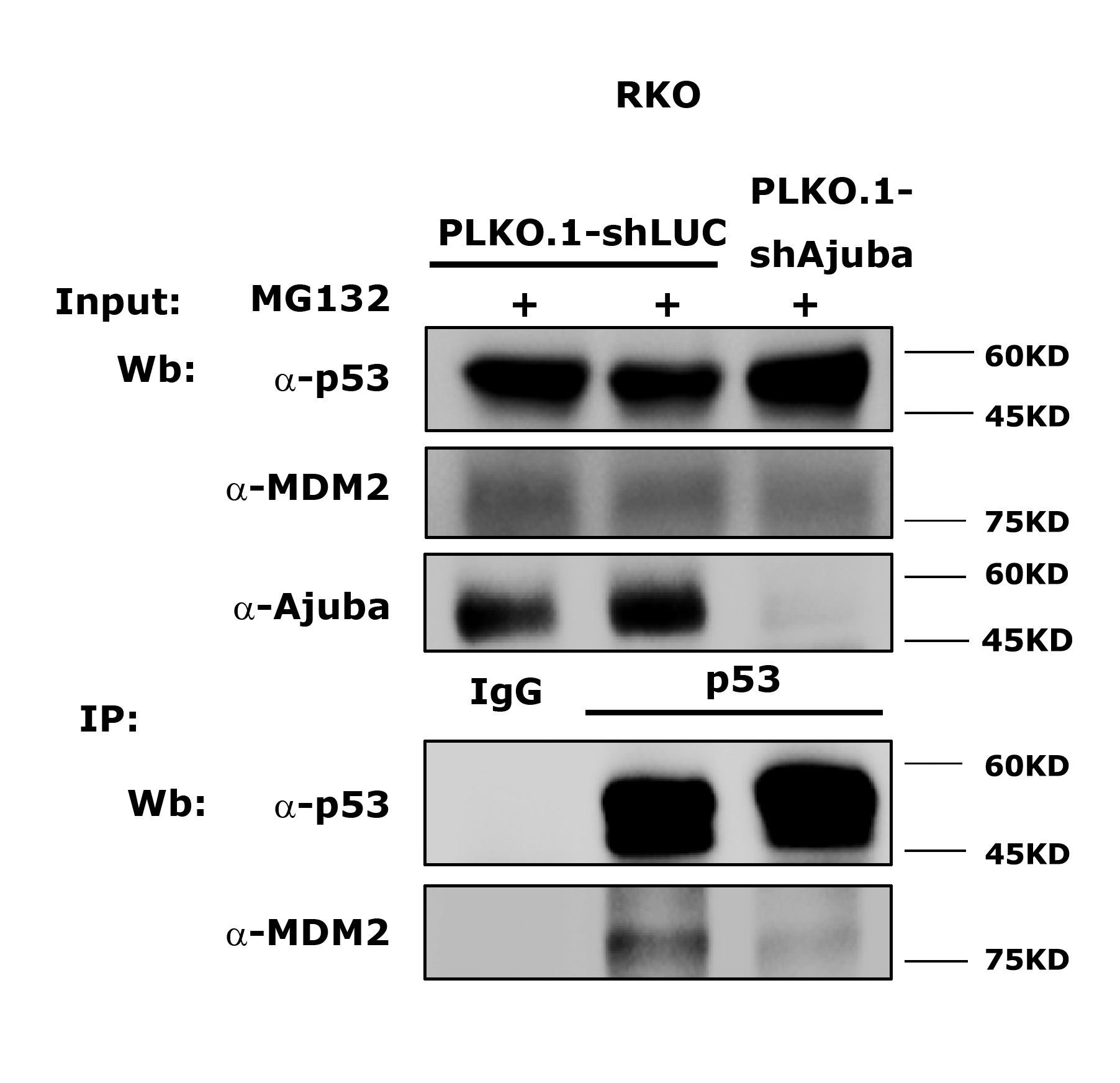

Supplement: Supplementary file 4 — Fig. S4. shAjuba inhibited the interaction between p53 and MDM2. RKO‐PLKO.1‐shAjuba and RKO‐PLKO.1‐shLUC cells were incubated with MG132 (10 μM) for 4–6 h before being harvested for endogenous co‐IP assay by using p53 antibody or normal control IgG. [file MOL2-17-1678-s005.jpg]

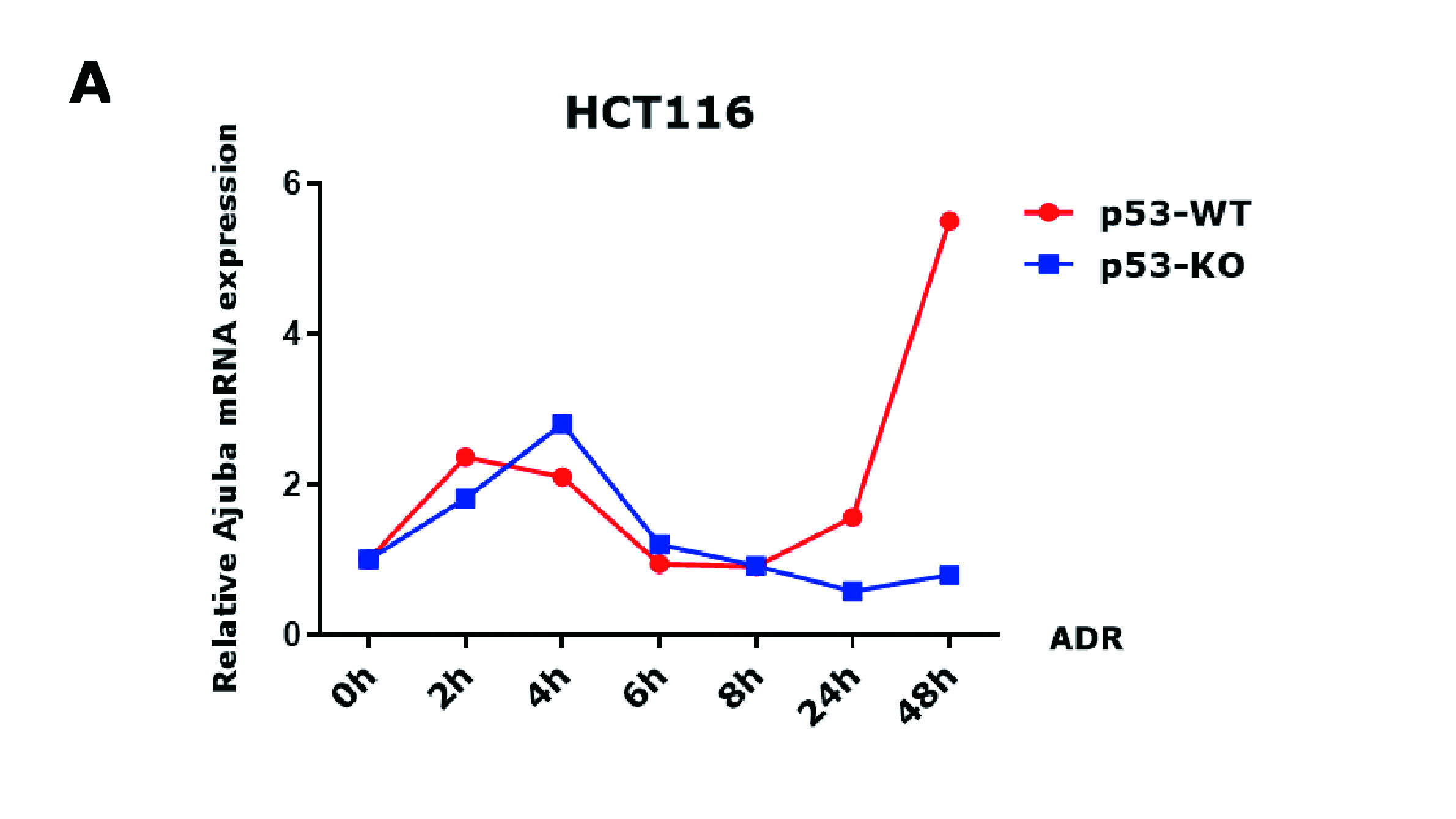

Supplement: Supplementary file 5 — Fig. S5. Ajuba expression can be induced by chemotherapy drugs. HCT116 p53+/+ or HCT116 p53−/− cells were incubated with 0.2μΜ Adriamycin (ADR) for indicated time and the RNA‐expression of Ajuba was detected by RT‐qPCR (data were shown as mean ± sd, three independent times). [file MOL2-17-1678-s004.jpg]
